# Reconstituted high-density lipoproteins promote wound repair and blood flow recovery in response to ischemia in aged mice

**DOI:** 10.1186/s12944-016-0322-4

**Published:** 2016-09-06

**Authors:** Tania Tsatralis, Anisyah Ridiandries, Stacy Robertson, Laura Z. Vanags, Yuen Ting Lam, Joanne T. M. Tan, Martin K. C. Ng, Christina A. Bursill

**Affiliations:** 1 The Heart Research Institute, 7 Eliza Street, Newtown, Sydney, 2042 Australia; 2 Sydney Medical School, University of Sydney, Camperdown, 2050 Sydney Australia; 3 Department of Cardiology, Royal Prince Alfred Hospital, Camperdown, 2050 Sydney Australia

**Keywords:** Aging, Vascular complications, High-density lipoproteins, Angiogenesis, Wound repair

## Abstract

**Background:**

The average population age is increasing and the incidence of age-related vascular complications is rising in parallel. Impaired wound healing and disordered ischemia-mediated angiogenesis are key contributors to age-impaired vascular complications that can lead to amputation. High-density lipoproteins (HDL) have vasculo-protective properties and augment ischemia-driven angiogenesis in young animals. We aimed to determine the effect of reconstituted HDL (rHDL) on aged mice in a murine wound healing model and the hindlimb ischemia (HLI) model.

**Methods:**

*Murine wound healing model—*24-month-old aged mice received topical application of rHDL (50 μg/wound/day) or PBS (vehicle control) for 10 days following wounding. *Murine HLI model*—Femoral artery ligation was performed on 24-month-old mice. Mice received rHDL (40 mg/kg) or PBS, intravenously, on alternate days, 1 week pre-surgery and up to 21 days post ligation. For both models, blood flow perfusion was determined using laser Doppler perfusion imaging. Mice were sacrificed at 10 (wound healing) or 21 (HLI) days post-surgery and tissues were collected for histological and gene analyses.

**Results:**

Daily topical application of rHDL increased the rate of wound closure by Day 7 post-wounding (25 %, *p* < 0.05). Wound blood perfusion, a marker of angiogenesis, was elevated in rHDL treated wounds (Days 4–10 by 22–25 %, *p* < 0.05). In addition, rHDL increased wound capillary density by 52.6 %. In the HLI model, rHDL infusions augmented blood flow recovery in ischemic limbs (Day 18 by 50 % and Day 21 by 88 %, *p* < 0.05) and prevented tissue necrosis and toe loss. Assessment of capillary density in ischemic hindlimb sections found a 90 % increase in rHDL infused animals. In vitro studies in fibroblasts isolated from aged mice found that incubation with rHDL was able to significantly increase the key pro-angiogenic mediator vascular endothelial growth factor (VEGF) protein (25 %, *p* < 0.05).

**Conclusion:**

rHDL can promote wound healing and wound angiogenesis, and blood flow recovery in response to ischemia in aged mice. Mechanistically, this is likely to be via an increase in VEGF. This highlights a potential role for HDL in the therapeutic modulation of age-impaired vascular complications.

## Background

Angiogenesis is an essential element in the regenerative response to tissue damage. It occurs in healing wounds and in response to ischemic injury. However, in advanced age, this process becomes impaired and is characterized by endothelial cell decline, altered inflammatory response and impaired neovascularization [1–3]. Consequently, impaired wound healing and disordered ischemia-mediated angiogenesis become defining features of age-impaired vascular complications. Aged individuals have higher mortality and increased rates of limb amputation as a result of acute limb ischemia [4]. In these circumstances, aged patients and animal models are reported to exhibit reduced tissue production of vascular endothelial growth factor (VEGF) and decreased hypoxia inducible factor (HIF)-1α stability and activation [5, 6]. Whilst this provides functional and mechanistic evidence for age-impaired wound healing and ischemia-mediated angiogenesis, the key triggers for these events have not been entirely elucidated. Recent data also indicate that processes fundamental to aging also play pivotal roles in cardiovascular disease associated with advanced age [7].

We have recently shown that high-density lipoproteins (HDL) augment ischemia driven angiogenesis in vivo and in vitro [8]*.* These effects in vitro were mediated via a stabilization of HIF-1α [9] and an elevation in VEGF [8]. We have also recently identified that reconstituted HDL (rHDL) rescues diabetes-impaired neovascularization [10]. Given the distinct mechanistic similarities between the impairment of angiogenesis in diabetes and in aging, we now seek to determine if rHDL can also improve age-impaired angiogenesis. We investigated this using two surgical murine models of wound healing and hindlimb ischemia and find that rHDL augments wound closure and hindlimb angiogenesis in aged mice. Clinical trials that have raised HDL in patients with advanced atheroma have been disappointing showing no benefit on advanced plaque size, which may be related to the age-induced failure of HDL function in the context of advanced atherosclerosis and aging. Our studies highlight an alternate therapeutic use of HDL for angiogenesis-related diseases that is effective in the context of aging, which may facilitate its eventual successful translation in clinic.

## Methods

### Preparation of discoidal reconstituted HDL

Apolipoprotein A-I (apoA-I) was isolated from plasma obtained from healthy humans by ultracentrifugation and anion-exchange chromatography, as described previously [11]. The collection of human blood complied with the ethical rules for human experimentation as stated in the 1975 Declaration of Helsinki. Ethics were obtained from the Sydney Local Health District Ethics Review Committee. Discoidal reconstituted HDL (rHDL) was prepared by cholate dialysis, complexing 1-palmitoyl-2-linoleoyl-phosphatidylcholine (PLPC) and apoA-I at an initial PLPC:apoA-I molar ratio of 100:1 (final PLPC:apoA-I molar ratio of 80:1). Protein concentration of rHDL was determined using bicinchoninic acid (BCA) assay (Thermo Scientific).

### Murine wound healing model

All experimental procedures and protocols were conducted with the approval from the Sydney Local Health District Animal Welfare Committee, and conformed to the Guide for the Care and Use of Laboratory Animals (United States National Institute of Health). Aged male 24-month-old C57Bl/6 J mice were used for both studies. The wound healing surgery was conducted as previously described [12]. Two full-thickness excisions that include the panniculus carnosus were created on the dorsum, one on each side of the midline of the mouse. A 0.5 mm thick silicone splint (Life Technologies, CA, USA) was then placed around the wound with adhesive and interrupted sutures. For each mouse, one wound received rHDL (50 μg/wound/day) and the other PBS (vehicle control), topically applied daily. A translucent occlusive dressing (Opsite™ Flexfix™, Smith & Nephew, London, UK) was then applied. Digital images were taken and micro-callipers were used to measure the wound area daily. Blood perfusion in the wound areas was determined using laser Doppler perfusion imaging (moorLDI2-IR, Moor Instruments, Devon, UK). Ten days after wounding, both wounds were excised for histological and gene analyses.

### Hindlimb ischemia model

The hindlimb ischemia model was conducted as described previously [8]. The left femoral artery and vein were ligated (7-0 silk) above both the epigastrica and profunda femoris before severing them distal to the ligation. The femoral artery and vein were also completely excised as distal as the popliteal from the hindlimb of mice. A sham procedure was performed on the opposite hindlimb. Mice received *i.v.* injections of PBS (vehicle control) or rHDL (40 mg/kg) every second day following surgery. Hindlimb blood reperfusion was determined by laser Doppler perfusion imaging (moorLDI2-IR, Moor Instruments) and performed prior to and immediately following surgery, then at Days 2, 4, 7, 10, 13, 16, 18 and 21 post-surgery. Following sacrifice, the gastrocnemius muscles of both ischemic and non-ischemic limbs were collected for histological and gene expression analyses.

### Immunohistochemistry

Wound tissues were fixed in paraformaldehyde (4 %) and embedded in paraffin. Wound sections (5 μm) were taken from the mid-point and assessed for the presence of Mac-3^+^ macrophages (1:100, Santa Cruz) and CD31^+^ neovessels (1:100, Abcam) [10]. Gastrocnemius muscle sections were frozen in OCT and fixed in 100 % methanol. Hindlimb sections (5 μm) were assessed for the presence of CD31^+^ neovessels (1:1000, Abcam), α-smooth muscle actin^+^ arterioles (1:500, Sigma Aldrich) and laminin (1:300, Merck Millipore) [10]. All images were analyzed using Image-Pro Plus 4.5 software (Diagnostic Instruments, USA). The percentage of positive staining was calculated for each high power field (minimum of 3 fields per section) and the mean staining area for each section calculated.

### Histochemistry

To determine collagen deposition, paraffin embedded wound sections were stained with Milligan’s trichrome stain. All images were analyzed using Image-Pro Plus 4.5 software (Diagnostic Instruments, USA). The percentage of positive staining was calculated for each high power field (minimum of 3 fields per section) and the mean staining area for each section calculated.

### qRT-PCR

Total RNA was isolated from wounds and hindlimbs with TRI reagent (Sigma Aldrich). The amount of RNA was quantitated spectrophotometrically using a NanoDrop^TM^ (Thermo Scientific). 200 ng total RNA was reverse transcribed using the iScript cDNA synthesis kit (Bio-Rad) in triplicate. All amplicons were amplified using iQ SYBR Green Supermix (Bio-Rad) in a Bio-Rad Cfx384 thermocycler and 20 pmol each of forward and reverse primer for murine VEGF (F: 5’-GGCTGCTGTAACGATGAAG-3’; R: 5’-CTCTCTATGTGCTGGCTTTG-3’), HIF-1α (F: 5’-TCCCTTGCTCTTTGTGGTTGGGT-3’; R: 5’-AACGTAAGCGCTGACCCAGG-3’), Siah1 (F: 5’-GACTGCTACAGCATTACCCACT-3’; R: 5’-GTTGGATGCAGTTGTGCCG-3’), Siah2 (F: 5’-CTAACGCCCAGCATCAGGAA-3’; R: 5’-GAACAGCCCGTGGTAGCATA-3’), PHD2 (F: 5’-ATCACCTGGATCGAGGGCAA- 3’; R: 5’-CGTTCGGCCGTTTATCCTGT-3’), PHD3 (F: 5’-GAGCCGGCTGGGCAAATACT-3’; R: 5’-GGGGTTGTCCACATGGCGAA -3’) and β2-microglobulin (B2M) measured as a reference gene (F: 5’-CAACGGCAGCATTTATAACCC-3’; R: 5’-CCCATTGATGATGGAGTGTGG-3’). Relative changes in mRNA levels of the genes of interest were normalized using the ^ΔΔ^CT method to murine B2M.

### ELISA

Wound and hindlimb tissue samples were homogenized with lysis buffer (80 mM Tris HCl, 10 mM NaCl, 50 mM NaF, 5 mM Na_4_P_2_O_7_, 15 nM Triton-X). Protein levels of mouse VEGF were determined from wound and hindlimb lysates using commercially available human VEGF ELISA kit (R&D Systems Inc).

### Plasma lipid concentration

Total cholesterol concentrations were determined enzymatically on mouse plasma using commercially available kits (Roche Diagnostics). HDL cholesterol concentrations were determined by enzymatic assay following precipitation of apolipoprotein B containing lipoproteins with polyethylene glycol. LDL levels were determined by subtracting total HDL from total cholesterol [8]. Triglyceride levels were determined enzymatically on mouse plasma using commercially available kits (Roche Diagnostics).

### In vitro studies

Fibroblasts isolated from aged mice (24 months) were grown to 80 % confluency in DMEM media in hypoxia (1 % O_2_/5 % CO_2_) and then incubated with either PBS (control) or rHDL (600 μg/ml) for 16 h [8]. Treated cells were lysed with radioimmunoprecipitation assay (RIPA) lysis buffer (20 mM Tris-HCl, 1 mM EDTA, 1 mM EGTA, 1 mM dithiothreitol, 0.5 mM phenylmethylsulfonyl fluoride, 1.5 μg/mL aprotinin, 1 μg/mL leupeptin, 1 μg/mL pepstatin, 1 mM sodium orthovanadate, and 0.2 % Triton X-100; pH 7.4). The protein concentration was determined using the BCA Protein Assay (Thermo Scientific, Waltham, MA, USA). Nitrocellulose membranes were incubated in a primary antibody against VEGF (1:1000, ab46154, Abcam, Cambridge, UK). Even protein loading was confirmed with α-tubulin (1:1000, ab40742, Abcam). Three donors were tested, each in quintuplicate (*n* = 15 total).

### Statistical analysis

Data are expressed as mean ± SEM. Differences between treatment groups were calculated using unpaired two tailed t-test or one-way ANOVA with Bonferroni’s comparison test *post hoc*. Significance was set at a two-sided *p* < 0.05.

## Results

### Topical rHDL promotes wound closure and wound angiogenesis in aged mice

**Fig. 1:**
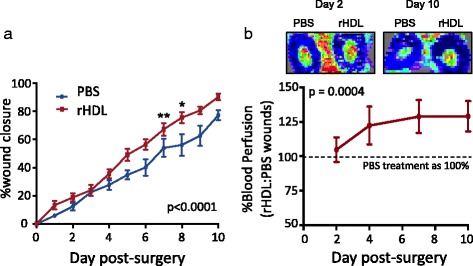
Topical rHDL promotes wound closure and wound angiogenesis in aged mice. Two full thickness wounds were created on the back flanks of 24-month-old C57Bl/6 J mice (*n* = 9/group). Mice received daily topical applications of rHDL (50 μg/wound) or PBS (vehicle). **a** Wound area was calculated from the average of three daily diameter measurements of the x, y and z-axes. Wound closure is expressed as a percentage of initial wound area at Day 0; **b** Blood flow perfusion was determined by laser Doppler imaging. Upper panels are representative Doppler images that depict high (*red*) to low (*blue*) wound blood flow taken at Days 2 and 10. Blood perfusion was determined based on rHDL:PBS wound ratio, with values above 100 % indicating increased blood perfusion with rHDL treatment relative to PBS control. Data is represented as mean ± SEM. Statistical analysis was performed by unpaired two-tailed t-test. *p < 0.05, **p < 0.01 vs. PBS treated wounds

We investigated the effect of topically applied rHDL in a murine model that mimics human wound repair in aged mice. Topical treatment of wounds with rHDL (50 μg/wound/day), in 24-month-old mice, showed significantly faster wound closure at Day 7 (25.6 %, *p* < 0.01) and Day 8 (22.2 %, *p* < 0.05) compared to PBS treated wounds (Fig. 1a). Consistent with this, blood perfusion determined by laser Doppler imaging, as a marker of wound angiogenesis, was significantly elevated in rHDL treated wounds from Days 4–10 (22–25 %, *P* < 0.05), compared to PBS control (Fig. 1b).

### The effect of rHDL on wound neovascularization, wound macrophage content and collagen deposition

**Fig. 2:**
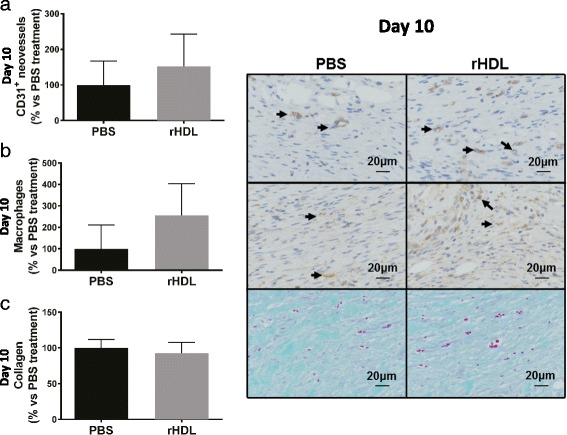
The effect of rHDL on wound neovascularization, wound macrophage content and collagen deposition. Two full thickness wounds were created on the back flanks of 24-month-old C57Bl/6 J mice (*n* = 9/group). Mice received daily topical applications of rHDL (50 μg/wound) or PBS (vehicle). On Day 10 post-wounding, wound tissue was collected and sectioned for: **a** Capillaries using immunohistochemistry for CD31^+^ (*brown*, denoted by *arrows*); **b** Macrophage infiltration was identified in wound sections using immunohistochemistry for Mac-3^+^ (*brown*, denoted by *arrows*); **c** Collagen deposition was determined using Milligan’s trichrome stain (*blue*). Photomicrographs represent wounds stained for CD31^+^, Mac-3^+^ and collagen of PBS and rHDL wounds following sacrifice. Data is represented as mean ± SEM

Wound sections were stained for CD31, a marker of neovessels. Despite a 52.6 % increase in CD31^+^ stained neovessels in rHDL treated wounds, there was no significant difference in the number of neovessels between wounds administered PBS or rHDL (Fig. 2a). Assessment of macrophage infiltration in wound sections with Mac-3 showed a non-significant increase in rHDL treated wounds (Fig. 2b). Finally, we found that there was no difference in collagen deposition between rHDL and PBS treated wounds (Fig. 2c).

### rHDL had no effect on gene expression of angiogenic markers in wounds

**Fig. 3:**
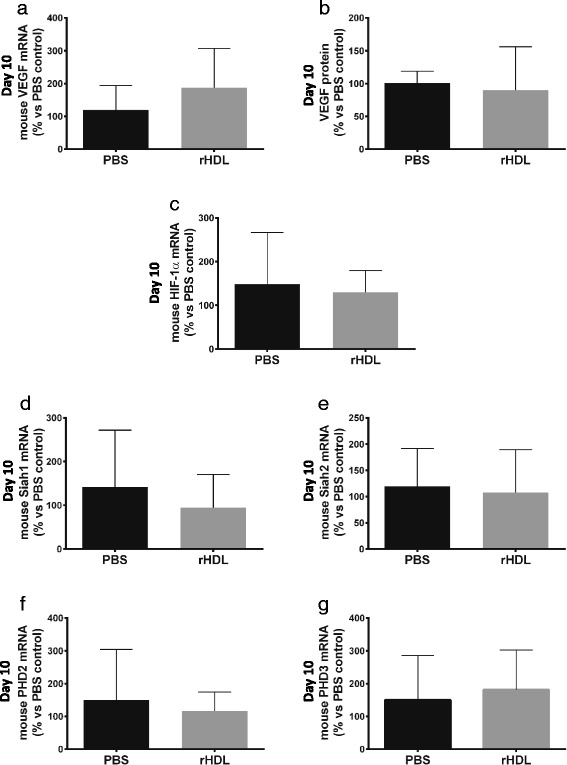
rHDL had no effect on gene expression of angiogenesis markers in wounds. Two full thickness wounds were created on the back flanks of 24-month-old C57Bl/6 J mice (*n* = 9/group). Mice received daily topical applications of rHDL (50 μg/wound) or PBS (vehicle). On Day 10 post-wounding, wound tissue was collected and RNA was isolated for qRT-PCR to assess gene expression or wound tissue was homogenized and used in ELISAs to assess protein. Measured markers include: **a** VEGF mRNA; **b** VEGF protein; **c** HIF-1α mRNA; **d** Siah1 mRNA; **e** Siah2 mRNA; **f** PHD2 mRNA; and **g** PHD3 mRNA. Data is represented as mean ± SEM

To elucidate the mechanism for the effects of rHDL on wound angiogenesis in aged mice, we examined the effect of rHDL on the key angiogenic growth factor vascular endothelial growth factor (VEGF). We found that topical application of rHDL had no impact on wound VEGF mRNA levels compared to PBS control wounds (Fig. 3a). Furthermore, treatment with rHDL did not affect VEGF protein levels in wounds (Fig. 3b). Hypoxia inducible factor (HIF)-1α, a key transcription factor that promotes VEGF expression in hypoxia, was not changed in wounds after topical rHDL treatment at Day 10 post-wounding (Fig. 3c). Examination of the post-translational modulators of HIF-1α found that there were no differences in the ubiquitin ligases Siah1 and Siah2 mRNA levels between rHDL and PBS treated wounds (Fig. 3d – e). Similarly, both proyl hydroxylases PHD2 and PHD3 showed no difference between rHDL and the PBS control group (Fig. 3f – g).

### rHDL promotes ischemia-induced blood perfusion in aged mice

**Fig. 4:**
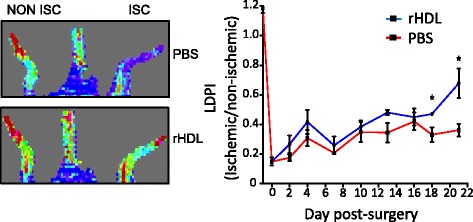
rHDL promotes ischemia-induced blood perfusion in aged mice. Femoral artery ligation was performed on 24-month-old C57B1/6 J mice (*n* = 6–8) to induce ischemia in hindlimbs. Mice received intravenous injections of PBS or rHDL (40 mg/kg) on alternate days following ligation until sacrifice 21 days later. Images represent high (*red*) or low (*blue*) blood flow in animals treated with PBS or rHDL at Day 21. Laser Doppler Perfusion Index (LDPI) was determined as a ratio of ischemic (ISC) to non-ischemic (NON ISC) hindlimbs. Data is represented as mean ± SEM. Statistical analysis was performed by unpaired two-tailed t-test. *p < 0.05 vs. PBS control

Laser Doppler perfusion imaging showed that blood flow was equally impaired across both treatment groups following ligation (Fig. 4). However, systemic rHDL infusions augmented blood flow in the ischemic hindlimbs at all time points, reaching significance at Day 18 (50 %, *p* < 0.05) and Day 21 (88 %, *p* < 0.05), compared to PBS infused control mice (Fig. 4).

### rHDL prevents tissue necrosis in ischemic hindlimbs of aged mice

**Fig. 5:**
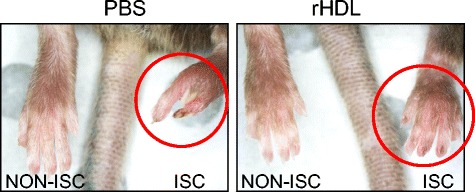
rHDL prevents tissue necrosis in ischemic hindlimbs of aged mice. Femoral artery ligation was performed on 24-month-old C57B1/6 J mice (*n* = 6–8) to induce ischemia in hindlimbs. Mice received intravenous injections of PBS or rHDL (40 mg/kg) on alternate days following ligation until sacrifice 21 days later. Images are representative mouse feet at Day 21

In support of our observations of improved blood flow in rHDL infused aged mice, we observed that in ischemic hindlimbs there was no loss of toes in mice that had been infused with rHDL (Fig. 5). In contrast, in the hindlimbs of control mice that had been infused with PBS, the loss of toes occurred in >60 % of mice. The loss of toes occurred in the third week post-ligation and is consistent with the failure of the control mice to mount a substantial blood flow recovery response. The ability of rHDL to prevent tissue necrosis is likely to be due to the promotion of blood flow perfusion in the final week in these mice.

### The effect of rHDL on hindlimb neovascularization

**Fig. 6:**
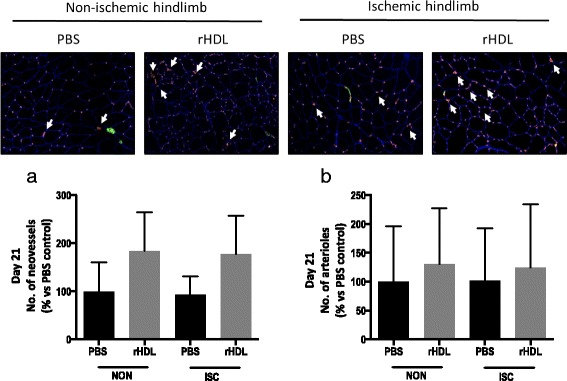
The effect of rHDL infusions on hindlimb neovascularization. Femoral artery ligation was performed on 24-month-old C57B1/6 J mice. Mice received i.v. injections of PBS or rHDL (40 mg/kg) on alternate days following ligation until sacrifice 21 days later. Upper panels are representative images of stained hindlimb sections at the 21-day time point. White arrows highlight capillaries. **a** Capillaries were identified in non-ischemic (NON) and ischemic (ISC) hindlimb sections using immunocytochemistry for CD31^+^ (*red staining*). (**b**) Arterioles were identified in non-ischemic and ischemic hindlimb sections using immunocytochemistry for α-actin^+^ (*green staining*). Data is represented as mean ± SEM

Next, we sought to determine the effect of rHDL on neovascularization by assessing capillary and arteriole density in ischemic and non-ischemic hindlimbs. Despite >90 % increases in CD31^+^ neovessels in both the ischemic and non-ischemic hindlimbs of rHDL infused mice, this did not reach significance when compared to control PBS infused mice (Fig. 6a). Assessment of α-actin^+^ arterioles revealed that there were very few arterioles and that rHDL infusions did not change arteriole number, in either ischemic or non-ischemic hindlimbs, compared to PBS control (Fig. 6b).

### The effect of rHDL on angiogenic markers in ischemic hindlimbs

**Fig. 7:**
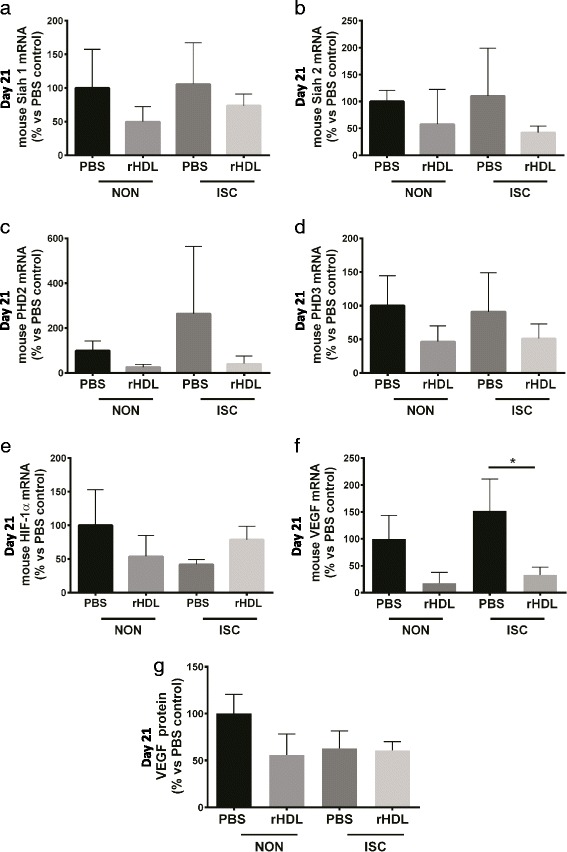
The effect of rHDL on angiogenic markers in ischemic hindlimbs. Femoral artery ligation was performed on 24-month-old C57B1/6 J mice. Mice received i.v. injections of PBS or rHDL (40 mg/kg) on alternate days following ligation until sacrifice 21 days later. On Day 21 post-surgery hindlimb tissue was collected and RNA isolated for qRT-PCR on non-ischemic (NON) and ischemic (ISC) hindlimbs to assess gene expression of (**a**) Siah1; (**b**) Siah2; (**c**) PHD2; (**d**) PHD3; (**e**) HIF-1α; and (**f**) VEGF, normalized to β2-microglobulin (B2M). **g** VEGF protein was determined on homogenized hindlimbs using ELISA. Data is represented as mean ± SEM. Statistical analysis was performed by one-way ANOVA and Bonferroni’s comparison test *post hoc*. *p < 0.05 vs. PBS controls

Examination of the post-translational HIF-1α modulators Siah1 and Siah2 (Fig. 7a – b) and PHD2 and PHD3 (Fig. 7c – d) showed no significant effects on mRNA levels in both ischemic and non-ischemic hindlimbs. rHDL treated mice showed a non-significant trend toward increased HIF-1α expression in ischemic hindlimbs (Fig. 7e). VEGF mRNA levels were unexpectedly lower in the ischemic hindlimbs of rHDL infused mice compared to PBS control, 21 days post-ligation (*p* < 0.05; Fig. 7f). Furthermore, when hindlimb VEGF protein levels were determined, no difference was found across both groups (Fig. 7g).

### rHDL increases VEGF in vitro in cells from aged mice

**Fig. 8:**
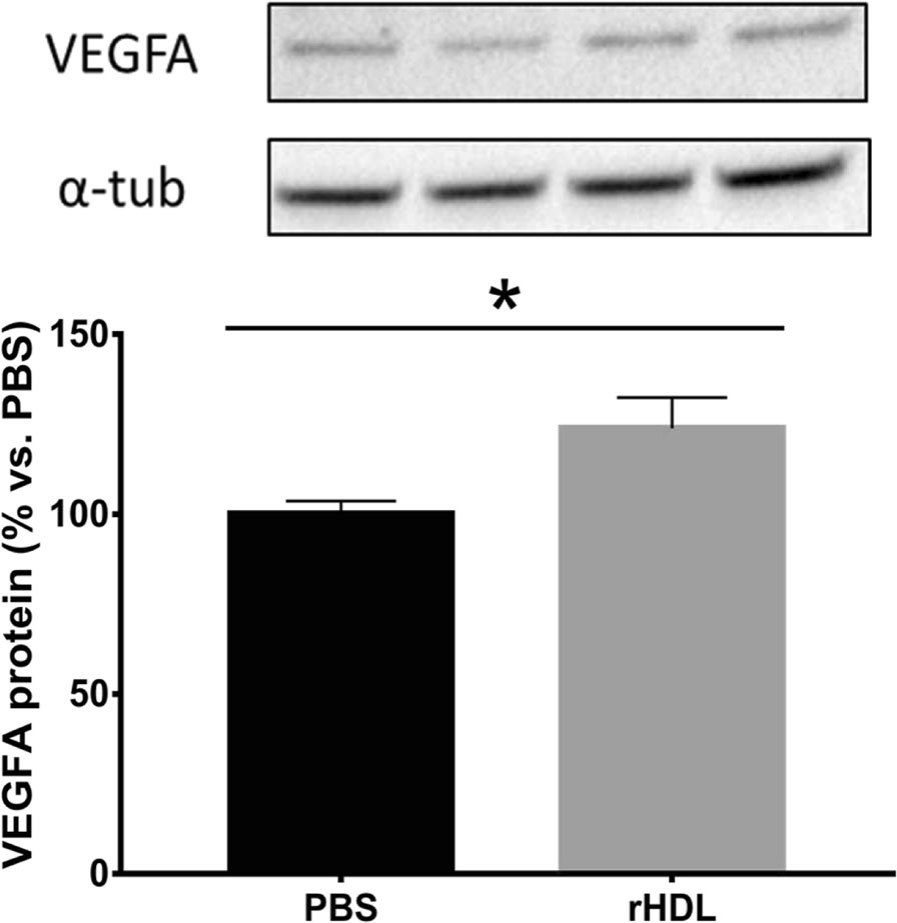
rHDL increases VEGF in fibroblasts from aged mice. Fibroblasts from aged mice were incubated with PBS (control) or rHDL for 16 h. VEGF protein was assessed on harvested cell lysates using western blotting. Cells from three donors were tested in quintuplicate (*n* = 15). Data is represented as mean ± SEM. *p < 0.05

Incubation with rHDL caused a significant increase (25 %, *p* < 0.0 %) in the key pro-angiogenic factor VEGF in fibroblasts isolated from aged mice (Fig. 8).

### rHDL infusions increase plasma total cholesterol and HDL cholesterol

**Fig. 9:**
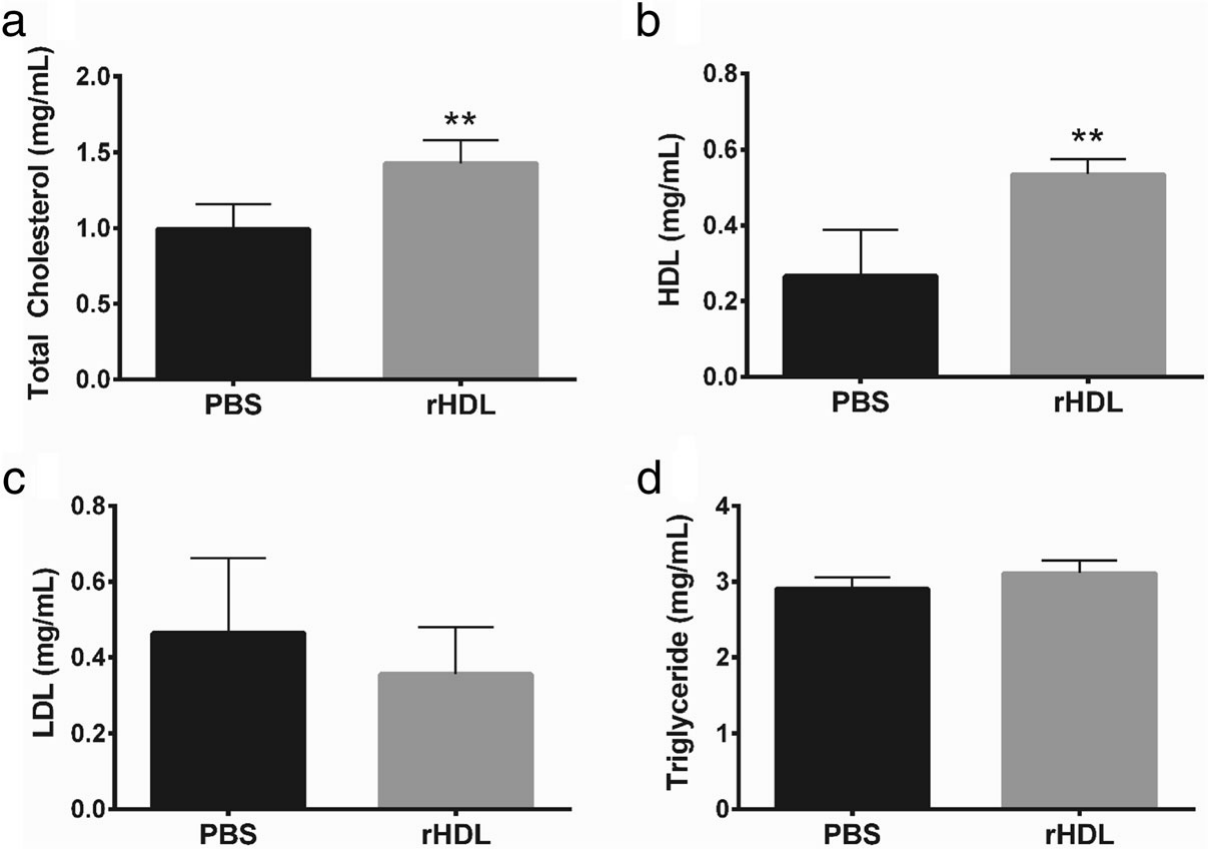
rHDL infusions increase plasma total cholesterol and HDL cholesterol. Femoral artery ligation was performed on 24-month-old C57B1/6 J mice. Mice received i.v. injections of PBS or rHDL (40 mg/kg) on alternate days following ligation until sacrifice 21 days later. Whole blood was collected via cardiac puncture at the 21 day time point and plasma isolated. **a** Total cholesterol concentrations were determined enzymatically on mouse plasma using commercially available kits; **b** HDL cholesterol concentrations were determined by enzymatic assay following precipitation of apolipoprotein B containing proteins with polyethylene glycol; **c** LDL levels were determined by subtracting total HDL from total cholesterol. **d** Triglyceride levels were determined enzymatically on mouse plasma using commercially available kits. Data is represented as mean ± SEM. Statistical analysis was performed by unpaired two-tailed t-test. **p < 0.01 vs. PBS control

Systemic rHDL infusions elevated both total cholesterol (*p* < 0.01; Fig. 9a) and HDL cholesterol (*p* < 0.01; Fig. 9b), compared to PBS infused control mice. rHDL did not affect plasma LDL or triglyceride levels (Fig. 9c – d). This increase in HDL indicates that the tail vein injections were delivered successfully.

## Discussion

Wound healing is a complex dynamic process and in healthy humans consists of four distinct, but overlapping, phases including: inflammation, granulation, re-epithelialization and tissue remodelling [13]. In advanced age, however, this process becomes disrupted, and is instead characterized by an altered inflammatory response, delayed re-epithelialization and impaired angiogenesis [14]. Impaired angiogenesis is also associated with age [15] and numerous studies utilizing subcutaneous implant and excisional models have shown delayed wound neovascularization in aged animals [16, 17]. The current study found that topical application of rHDL improved wound repair and wound angiogenesis in aged mice. Furthermore, systemic delivery of rHDL augmented blood flow recovery and prevented tissue necrosis in response to ischemia. Mechanistically these changes may be mediated through an increase in VEGF. These studies show the efficacy of HDL in the rescue of aged-impaired wound repair and angiogenesis.

The potent vasculo-protective properties of HDL are well known [18] and previous studies from our lab have shown that in young mice rHDL augment wound closure and ischemia-driven angiogenesis [8, 19]. This is the first study to show that rHDL can still effectively improve wound closure and angiogenesis in aged mice in which there are impairments in normal healing responses. Recent (and current) clinical trials that have raised HDL have all been aimed at reducing advanced atherosclerotic plaques in elderly individuals [20–23]. These studies have been disappointing, as they have shown no cardiovascular benefit despite increases in HDL of 11–70 %. This puts into question HDL raising as a therapy to reduce advanced plaque. A number of mechanisms may contribute to this failure including the reduced ability of HDL to efflux cholesterol from advanced necrotic plaques in aged individuals [24]. Furthermore, studies have found that the anti-atherogenic and anti-oxidant properties of HDL become compromised with aging and in patients with advanced coronary artery disease (CAD). HDL isolated from elderly individuals induces less macrophage cholesterol efflux and is less able to inhibit LDL oxidation when compared to HDL from young individuals [24]. HDL isolated from elderly individuals is more susceptible to oxidation than HDL isolated from young individuals [25], which has been attributed to age-related decreases in PON1 [26]. HDL from CAD patients fails to activate eNOS or NO. This has also been attributed to reductions in HDL-associated PON1 [26]. Finally, oxidatively modified apoA-I has impaired cholesterol efflux, is pro-inflammatory and is abundant in established plaques [27, 28]. Collectively, these studies suggest HDL functionality is compromised when raised in an aged/diseased milieu in the context of CAD and perhaps dietary antioxidant intake could counteract this, although the data are still conflicting as to their effectiveness [29]. Our studies, however, clearly show that if targeted to the right pathology such as wound repair and impaired ischemia-mediated angiogenesis, rHDL can still be strikingly effective in the context of aging. These findings may be important for the targeting of the HDL raising interventions in future clinical studies.

To further examine the impact of topical rHDL treatment, wounds were assessed for markers of angiogenesis and wound repair. The initial inflammatory phase of wound healing is distinguished by the infiltration of macrophages, which release pro-inflammatory growth factors such as VEGF, to promote vascular permeability and fibroblast activity. Age related alterations in wound repair are marked by delayed macrophage infiltration in young and middle-aged mice [30]. The next phase of wound healing is characterized by angiogenesis (capillary growth), granulation and ultimately collagen synthesis [31]. We found that there were non-significant increases in wound capillaries and macrophages. The lack of significant effect is likely because the increase in the inflammatory response and requirement for angiogenesis post-wounding occurs in the early stages of wound repair. Our histological analysis was performed on wound sections from mice at sacrifice (Day 10), a time point at which the importance of these repair processes are declining. Another contributing factor to the lack of significant increase in wound macrophage content is that HDL has potent anti-inflammatory properties and has been shown to reduce macrophage infiltration in atherosclerotic plaques [32]. This anti-inflammatory effect of HDL may therefore have counteracted any increases in wound macrophage content as part of the repair process. We also found no changes in wound collagen content between PBS and rHDL treated wounds. Collagen composition in old mice does not differ to that in young mice, however, in mature wounds there is decreased collagen deposition associated with age [15]. Our studies suggest that rHDL has no effect on the ability of a wound to deposit collagen.

VEGF is a key contributor to multiple components of the wound healing cascade including angiogenesis, epithelialization and collagen deposition [33]. Reduced levels of VEGF are associated with delayed capillary growth [15] and VEGF replacement can reverse impaired angiogenesis in aged mice [34]. Hypoxia-inducible factor-1α (HIF-1α) is induced in ischemic conditions to promote angiogenesis and in turn promote the expression of potent angiogenic factors such as VEGF. Furthermore, HIF-1α is also post-translationally modulated. Upon hypoxic stimulation, the E3 ubiquitin ligases Siah1 and Siah2 are induced which suppress the expression of prolyl hydroxylases (PHDs) PHD2 and PHD3. Under normoxic conditions, the PHDs induce the ubiquitination and degradation of HIF-1α by hydroxylating specific prolyl residues. Studies from our laboratory have shown that HDL augments ischemia driven angiogenesis in vivo and in vitro [8] and that these effects are mediated via the post-translational stabilization of HIF-1α [9]. Based on our previous findings with HDL and as rHDL treatment increased the rate of wound closure and wound angiogenesis in aged mice in the current study, it may have been expected that there would have been elevations in both VEGF and HIF-1α in the tissues of the animals. We did not, however, find any differences in these key angiogenic factors. The lack of change may be due to the time point at which the wounds were analyzed. VEGF is induced early in the wound healing process to stimulate wound angiogenesis and promote healing. Following this initial induction, VEGF levels then return to baseline. This is consistent with the lack of change observed in these angiogenic markers in our study at the Day 10 time point. It is also consistent with the increase VEGF in the aged fibroblasts following incubation with rHDL in our in vitro studies.

In addition to the deleterious impact that aging has on wound healing, studies have also shown that angiogenesis is responsible for collateral development (such as that seen in peripheral limb ischemia) and is impaired with aging, often leading to amputation [35]. Neovascularization is a critical part of the recovery process after ischemia [36] and aged patients show decreased capillary formation in response to ischemia [37, 38]. We have recently shown that HDL can augment ischemia-mediated angiogenesis in young mice [8]. The current study has now found that HDL can augment angiogenesis in the hindlimb ischemia model in aged animals. In the aged mice we observed that blood flow recovery was slower than in our previous work in young mice. In young mice blood perfusion starts to increase at 4–6 days post-femoral artery ligation and at 10–14 days blood perfusion levels are at ~80 % of the original blood flow before the ligation [8]. In the aged mice infused with PBS, blood flow recovery does not go above 40 % for the entire experimental period of 21 days. Only in mice infused with rHDL does blood flow increase to 50–70 % in the final experimental week. This suggests that rHDL is able to overcome age-impaired neovascularization in response to ischemia. In support of our observations of improved blood flow in rHDL infused aged mice, we observed that in ischemic hindlimbs there was no loss of toes in mice that had been infused with rHDL. The ability of rHDL to prevent tissue necrosis and toe loss is likely due to the promotion of blood flow perfusion in the final week in these aged mice. There was also a >90 % increase in hindlimb CD31^+^ neovessels which is consistent with the increase in blood perfusion and protection against tissue necrosis in rHDL infused mice. We did not, however, see any changes in arterioles. Very few arterioles were detected in the hindlimb sections of these aged mice and perhaps a reflection of the delay in neovascularization and subsequent lack of maturation into arterioles by the Day 21 time point.

We have previously found that rHDL augments VEGF levels in ischemic hindlimbs in young mice [8]. This study, however, found that mRNA levels of VEGF were significantly lower in the hindlimbs of mice infused with rHDL and there was no change in hindlimb VEGF protein. The end points in our previous studies was 10 or 14 days. It is possible therefore that our results for VEGF expression in aged mice may indicate that rHDL-induced increases in hindlimb VEGF occur earlier post-ligation than at the 21 day time point at which they were measured.

## Conclusions

In conclusion, in aged mice rHDL increased wound closure and wound angiogenesis, and augmented blood perfusion in ischemic hindlimbs, preventing tissue necrosis. These studies demonstrate the efficacy of rHDL for the treatment of angiogenesis-related diseases that are associated with aging and highlight an alternate therapeutic pathway for the translation of HDL to clinic.

## Abbreviations

apo A-I: Apolipoprotein A-I
HDL: High density lipoproteins
HIF-1α: Hypoxia inducible factor 1-α
HLI: Hindlimb ischemia (model)
PBS: Phosphate buffered saline
PHD: Proyl hydroxylase
rHDL: Reconstituted HDL
VEGF: Vascular endothelial growth factor
